# Endocannabinoid and steroid analysis in infant and adult nails by LC–MS/MS

**DOI:** 10.1007/s00216-022-04189-y

**Published:** 2022-07-04

**Authors:** Tanja Restin, Nastassja Byland, Clarissa D. Voegel, Pearl La Marca-Ghaemmaghami, Markus R. Baumgartner, Dirk Bassler, Thomas Kraemer, Tina M. Binz

**Affiliations:** 1 Newborn Research Zurich, Department of Neonatology, University Hospital Zurich and University of Zurich, Zurich, Switzerland; 2 Institute of Physiology, University of Zurich, Zurich, Switzerland; 3 Center for Forensic Hair Analytics, Zurich Institute of Forensic Medicine, University of Zurich, Zurich, Switzerland; 4 Psychology Counselling and Research Institute for Sexuality, Marriage and the Family, International Academy for Human Sciences and Culture, Walenstadt, Switzerland; 5 Department of Forensic Pharmacology and Toxicology, Zurich Institute of Forensic Medicine, University of Zurich, Zurich, Switzerland

**Keywords:** Stress, Long-term monitoring, Nails, Endocannabinoids, Steroids, LC–MS/MS

## Abstract

A common method to quantify chronic stress is the analysis of stress markers in keratinized matrices such as hair or nail. In this study, we aimed to validate a sensitive liquid chromatography–tandem mass spectrometry (LC–MS/MS) method for the combined quantification of steroid hormones and endocannabinoids (eCBs) in the keratinized matrix nail. Furthermore, we aimed to investigate the suitability of the nail matrix for the detection of these stress markers in a pilot study. An LC–MS/MS method was used for the simultaneous identification and quantification of four eCBs (2-arachidonoylglycerol (2-AG), anandamide (AEA), oleoylethanolamide (OEA), palmitoylethanolamide (PEA)) and five steroid hormones (cortisol, cortisone, androstenedione, progesterone, testosterone) in human nails using a surrogate analyte method for each analyte. The method was validated in terms of selectivity, response factor, linearity, limit of quantification (LOQ), precision, accuracy, matrix effect, recovery, robustness, and autosampler stability. Nail samples were extracted for 1 h with methanol following a clean-up with a fully automated supported liquid extraction (SLE). The influence of nail weight on the quantification was investigated by using 0.5–20 mg of nail sample. As a proof of concept, nail samples (*N* = 57) were analyzed from a cohort representing newborns (1 month old), children (between 1 and 10 years), and adults (up to 43 years). It could be shown that the established workflow using a 1 hour extraction and clean-up by SLE was very robust and resulted in a short sample preparation time. The LC–MS/MS method was successfully validated. Matrix effects with ion enhancement occurred mainly for 2-AG. Sample weights below 5 mg showed variations in quantification for some analytes. Certain analytes such as PEA and progesterone could be accurately quantified at a sample weight lower than 5 mg. This is the first study where steroids and eCBs could be simultaneously detected and quantified in infant and adult nails. These results show that nails may serve as an alternative keratinized matrix (compared to hair) for the retrospective monitoring of cumulative eCB and steroid hormone levels. The combined assessment of eCBs and steroids from nails could provide a new approach to gain new insights into stress exposure in newborns and adults.

**Graphical abstract:**

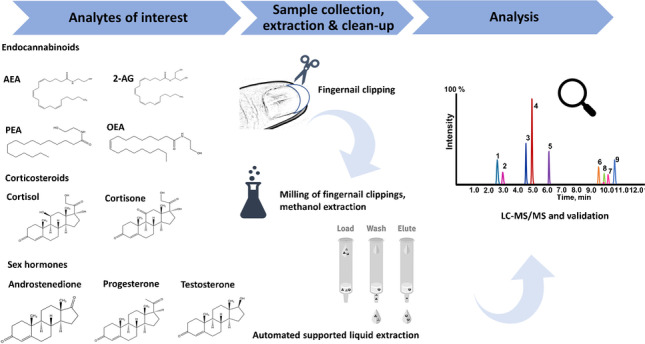

**Supplementary Information:**

The online version contains supplementary material available at 10.1007/s00216-022-04189-y.

## Introduction

Stress activates amongst other systems the hypothalamic–pituitary–adrenal (HPA) axis, which is a key regulator in numerous physical and psychological reactions [1, 2]. Cortisol, the end product of the HPA axis, is one of the most prominent stress hormones in humans, and is currently known as a valid biomarker for stress [3].

The stress response is balanced through the endocannabinoid system, which acts against the stress emotions of anxiety, anger, and fear [4]. The endocannabinoid system is a neuromodulatory system consisting of cannabinoid (CB) receptors and their endogenous ligands [5]. Stress exposure may change the concentrations of the two main endocannabinoid (eCB) molecules anandamide (AEA) and 2-arachidonoylglycerol (2-AG) which are mediated by CB1 and CB2 receptors [6, 7]. ECBs support the adaptation to a challenging environment, increase tissue regeneration, and thus have been hypothesized to play an important role in processes involved in stress adaptation and resilience [8]. It seems that OEA and PEA concentrations change under chronic stress and could therefore play a key modulatory role in stress resilience [9–11]. Blood, urine, and saliva are most commonly used to monitor stress hormones as reviewed by Lewis [12]. However, such methods have the disadvantage that the hormone concentrations change during the day according to their circadian rhythms [13]. In addition, these matrices are subject to high individual fluctuation and prone to bacterial overgrowth [14, 15]. Alternative matrices that can reflect cumulative steroid levels over a longer period of time are hair and nails [16, 17]. For instance, Tegethoff et al. analyzed fetal steroid exposure in utero from infant nails and showed that determination of fetal stress biology in nails is possible [18]. However, while steroids have been determined in nails [16, 18, 19], eCBs have not been previously analyzed in this matrix. The measurement of very low endogenous steroid and eCB concentrations from keratinized matrices such as hair and nails presents analytical challenges. Nowadays, sensitive liquid chromatography–tandem mass spectrometry (LC–MS/MS) is mainly used for the quantification of eCBs and steroid hormones in hair [17, 20]. The lack of real analyte-free matrices makes the quantification of endogenous compounds complex and challenging. Common approaches that are used for the quantification of endogenous compounds are standard addition, surrogate matrix, or surrogate analytes [21]. The approach of surrogate analytes has previously been used to overcome the problem of having no analyte-free hair matrix for the quantification of steroid hormones [16, 22, 23]. A surrogate analyte is a stable isotope–labeled analyte, which does not occur naturally in the samples. It has the same physicochemical properties as the endogenous compound, and thus, a calibration with the surrogate analyte can be used for quantification.

The present study aimed to validate a sensitive LC–MS/MS method for the simultaneous quantification of eCBs and steroid hormones in human fingernail clippings using surrogate analytes. The aim was to find a suitable and simple sample preparation for nails to simultaneously analyze a panel of endogenous stress hormones including steroids and eCBs and prove the applicability of the method.

## Materials and methods

**Scheme 1:**
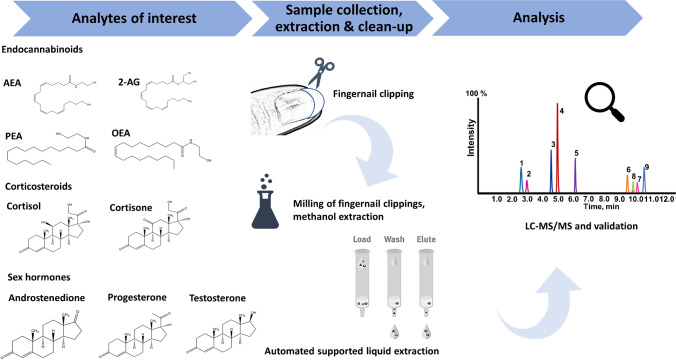
Workflow of sample preparation and analytical measurement for the combined analysis of steroids and endocannabinoids in nails

The analytical procedure is summarized in Scheme 1.

### Chemicals

OEA, PEA, the deuterated standards D_9_-progesterone and D_7_-cortisone, and ^13^C-labeled standards ^13^C_3_-androstenedione, ^13^C_3_-testosterone, and ^13^C_3_-progesterone were purchased from Sigma-Aldrich (Buchs, Switzerland). ^13^C_3_-cortisol and ^13^C_3_-cortisone were purchased from Isoscience (Ambler, USA). AEA, 2-AG, and the deuterated eCBs (D_4_-AEA, D_5_-2-AG, D_4_-OEA, D_4_-PEA, and D_11_-AEA) were purchased from Cayman Chemicals (Ann Arbor, USA). Water and methanol (MeOH) were of LC–MS grade (Chromasolv®) and purchased from Sigma-Aldrich (Buchs SG, Switzerland). Acetone, ethyl acetate, and ammonium fluoride were purchased from Merck (Darmstadt, Germany). Reconstitution solution consisted of 0.2 mM NH_4_F in water/methanol 97/3 v/v, respectively. Isolute® SLE + columns were purchased from Biotage® (Uppsala, Sweden). All chemicals were of highest analytical grade.

### Preparation of standard stock solutions

Final standard concentrations of 1 ng/µL of each analyte were prepared in methanol for the steroid hormones and in acetonitrile for the eCBs. The internal standard mixture (D_7_-cortisone, D_9_-progesterone, D_11_-AEA) was prepared in acetonitrile at a final concentration of 40 pg/µL for D_9_-progesterone and D_11_-AEA and 80 pg/µL for D_7_-cortisone. For calibration and validation experiments, stock solutions were prepared in different concentrations: 0.2, 2, 4 and 20 pg/μL for D_4_-AEA and ^13^C_3_-testosterone; 0.2, 2, 20 and 200 pg/μL for ^13^C_3_-cortisone, ^13^C_3_-androstenedione, and ^13^C_3_-progesterone; 0.2, 2 and 20 pg/μL for ^13^C_3_-cortisol; 2, 20 and 200 pg/μL for D_5_-2-AG and 2, 200, 1000, and 10,000 pg/μL for D_4_-PEA and D_4_-OEA. The stock solutions were used to spike nail samples for calibration and quality control (QC) samples. All stock solutions were stored at – 20 °C until use. A summary of analytes, surrogate analytes, and internal standards can be found in Table S1 in Supplemental.

### Nail sample preparation and extraction

Nails for validation and proof of principle were collected from volunteers who gave verbal consent. For children’s nails, we received the informed consent of the parents. The present study was performed in full accordance with Swiss laws, particularly those pertaining to the use of human materials in research. A statement of the Ethics Board of the Canton of Zurich (document BASEC-no. Req-2017–00946) was obtained. For validation experiments, several different nail pools from healthy volunteers were prepared as follows: nails were pooled from different volunteers, transferred into 5 mL glass vials and washed for 3 min with 2 mL deionized water, followed by washing for 2 min with the same amount of acetone. The washing was done to remove surface contamination from the nail samples. The vials were shaken by hand during the washing process. The washing solutions were decanted and disposed, and the nail samples were dried overnight at room temperature. For preparation of nail pools, the dried nail samples were homogenized in a steel cylinder (20 mm diameter) for 10 min at 30 Hz with one milling ball, resulting in a fine powder. For validation, different amounts (0.5 to 20 mg) of pulverized nail samples were used for the different experiments (details for validation and the pools can be found in Supplemental). For the authentic samples, 10–20 mg of nail clippings were weighted into a 1.5-mL Eppendorf tube after washing and drying and 3 milling balls (stainless steel, diameter 5 mm) were added. The exact amount of nail that was used per sample is indicated in Table S2. For some samples, less than 10 mg was used. For pulverization, the samples were milled for 10 min at a frequency of 30 Hz. For extraction, 50 μL internal standard mixture and 1 mL methanol were added to the pulverized nail sample. The samples were briefly shaken and placed in a sonication bath for 1 h (35 kHz, 600 W) at a temperature of 55 °C for extraction. The extract was transferred into a column rack (24 × 6 mL) from Biotage® Extrahera (Biotage, Uppsala, Sweden) for supported liquid extraction (SLE). Sample extracts were automatically loaded onto Isolute SLE + columns and allowed to absorb for 5 min. Analytes were then eluted two times with 2.5 mL ethyl acetate with a wait time of 5 min in between. The extracts were dried in a Turbovap® (Biotage, Uppsala, Sweden) with a set bath temperature of 35 °C for 1 h and resuspended in 60 μL MeOH and 140 μL reconstitution solution (eluent B). For extraction efficiency, varying extraction times and nail weights were tested (see “Results” section).

### Method validation

The validation was performed in accordance with the guidelines of the Society of Toxicological and Forensic Chemistry (GTFCh) [24]. 20 mg of a nail pool was used for the validation experiments and QC samples. The following parameters were evaluated: response factor (for the surrogate analytes), selectivity, linearity, limit of quantification (LOQ), accuracy, precision, matrix effect, recovery, robustness, and autosampler stability. For validation, the approach of using a surrogate analyte was applied as described before [16, 22, 25, 26]. Four deuterated standards were chosen as surrogate analytes for the eCBs (D_4_-AEA, D_5_-2-AG, D_4_-OEA, and D_4_-PEA), and five stable isotope–labeled standards were chosen for the steroid hormones (^13^C_3_-cortisol, ^13^C_3_-cortisone, ^13^C_3_-androstenedione, ^13^C_3_-progesterone, ^13^C_3_-testosterone). The internal standard contained D_7_-cortisone, D_9_-progesterone, and D_11_-AEA. For all surrogate analytes, the response factor was calculated by the ratio of the responses found for the surrogate and authentic analytes. If the response factor was not 1, it was incorporated into the regression line of the calibration curve in order to get the correct result for the quantification of the endogenous analyte. The selectivity was determined in nail matrix for the surrogate analytes and in neat solutions for all analytes. Therefore, unspiked “blank” nail samples were extracted and the transitions of the surrogate analytes were evaluated for interferences. Furthermore, 25 µL of 1 ng/µL standard solutions of analytes and surrogate analytes were mixed separately with 350 µL mobile phase A and analyzed. For each sample, the different transitions were evaluated for possible cross-interferences of the transitions or with matrix. In general, validation parameters for steroid hormones and eCBs were determined as follows: for linearity, seven calibrators with increasing concentrations and an unspiked sample were prepared. The calibration range for D_5_-2-AG was 5 to 200 pg/mg; for D_4_-AEA and ^13^C_3_-testosterone 0.1 to 10 pg/mg; for D_4_-OEA 20 to 10,000 pg/mg; for D_4_-PEA 500 to 10,000 pg/mg; for ^13^C_3_-cortisol 0.3 to 50 pg/mg; for ^13^C_3_-cortisone 1 to 500 pg/mg; for ^13^C_3_-androstenedione 0.1 to 500 pg/mg and for ^13^C_3_-progesterone 0.3 to 500 pg/mg. The regression lines (*R*^2^) were calculated for D_4_-OEA and D_4_-PEA with a quadratic model. For the other surrogate analytes, a simple linear model with 1/*x* weighting and no weighting for eCBs was used. The LOQ was calculated and determined applying signal-to-noise ratios of 10:1 or higher, respectively. For accuracy and precision, duplicates of a nail pool with different end concentrations (low, medium, and high concentration ranges) according to substance classes were prepared by spiking with different stock solutions. The measurements were carried out on six consecutive days. The bias as well as the intra- and inter-day precision was calculated for each analyte in each matrix. For matrix effect and recovery, six replicates from a nail pool at two different concentration levels (low and high) were analyzed. For the matrix effect, the ratio of peak areas of spiked nail (*A*) to spiked solvent (*B*) at the same concentration was compared (matrix effect = (*A*/*B*) × 100). For recovery, the ratio of the peak area of spiked matrix before (*C*) and after (*D*) extraction was compared (recovery = (*C*/*D*) × 100). For robustness, six replicates of an authentic nail pool were analyzed and the mean value of detectable analytes plus the relative standard deviation was determined. For stability, six spiked QC samples at low, medium, and high concentration levels (same as accuracy and precision) were prepared and pooled for each concentration level. The pools were aliquoted and placed in the autosampler (4 °C) at different positions in the time frame representing a standard batch (24 samples). Furthermore, an authentic pool was prepared and also measured in the same way.

### LC–MS/MS analysis

An LC–MS/MS device was used for the analysis of the sample extracts as described in a previous publication [22]. In brief, separation of the analytes was carried out with a Prominence UFLC system (Shimadzu, Kyoto, Japan) by injecting 10 µL of the samples onto a Phenomenex® Kinetex® XB-C_18_ (2.6 µm, 50 × 2.10 mm) column. The mobile phase consisted of 0.2 mM NH_4_F in water/methanol 97/3 v/v respectively (*A*) and 0.2 mM NH_4_F in water/methanol 3/97 v/v (*B*), and the flow rate was 0.40 mL/min. The column oven temperature was kept at 40 °C. The following gradient was applied: 0–40% *B* for 0–0.1 min, isocratic 40% from 0.1 to 1 min, 40–70% *B* from 1 to 5 min, isocratic 70% for 5–6 min, 70–80% from 6 to 7 min, isocratic 80% for 7–11 min, 80–90% from 11 to 14 min, isocratic 99% *B* from 14.1 to 17 min, 99–40% *B* from 17 to 19 min followed by an equilibration step of 1 min. Analyte detection was performed using a QTRAP® 6500 + linear ion trap quadrupole mass spectrometer (Sciex®, Darmstadt, Germany) with an electrospray ionization (ESI) source. Positive ESI mode and multiple reaction monitoring mode (MRM) with an ion spray voltage of 4500 V were used for quantification. The method parameters are listed in Table S3 in Supplemental. The curtain gas was fixed at 20 psi, the collision gas was set to high, and the ion source gases 1 and 2 were at 70 psi and 50 psi, respectively. The source temperature was set to 600 °C. Analyst® software (version 1.6.3, Sciex, Darmstadt, Germany) was used for instrument control and data analysis.

### Statistical analysis

For statistical analysis, GraphPad Prism 7.0 (GraphPad Software, San Diego, CA, USA) was used. The Shapiro–Wilk normality test revealed that the data were not normally distributed. Group comparisons were conducted using the Wilcoxon signed-rank test for paired samples and Mann–Whitney test for unpaired samples. Correlation coefficients were assessed using Spearman correlation. *p* values > 0.05 were considered as not statistically significant (ns), *p* < 0.05 (*) as significant, *p* < 0.01 (**) as very significant, and *p* < 0.001 (***) and *p* < 0.0001 (****) as extremely significant.

## Results

### Analytical LC–MS/MS method

**Fig. 1:**
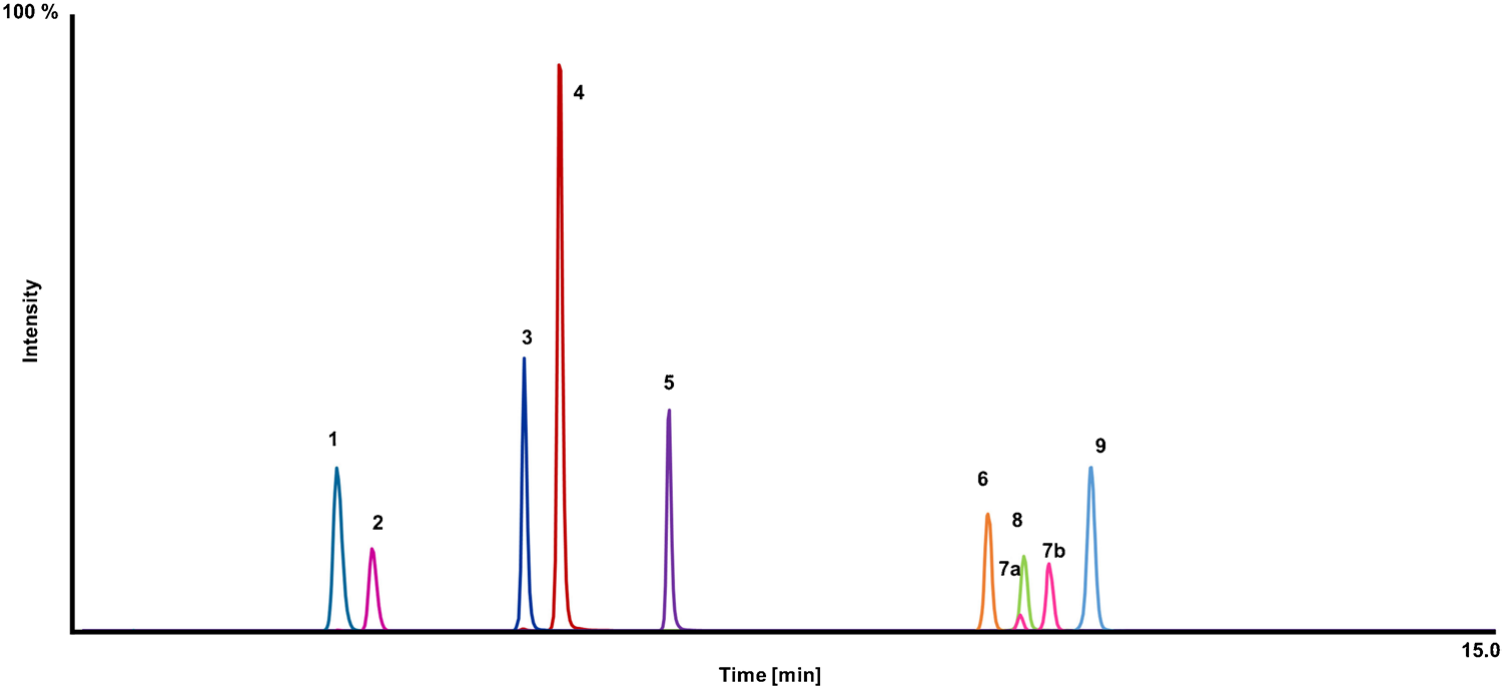
Final chromatogram of the scheduled LC–MS/MS method obtained from a neat solution mix of 1 ng/μL. 1 = ^13^C_3_-cortisone, 2 = ^13^C_3_-cortisol, 3 = ^13^C_3_-androstenedione, 4 = ^13^C_3_-testosterone, 5 = ^13^C_3_-progesterone, 6 = D_4_-AEA, 7a/b = D_5_-1-AG/2-AG, 8 = D_4_-PEA, 9 = D_4_-OEA

An existing LC–MS/MS method for detection of steroids and eCBs in hair was used as a starting point for method validation [22]. The method was adapted by replacing eCB analytes with deuterated analytes to apply the approach of surrogate analytes for each authentic analyte. The chromatographic conditions remained unchanged. For steroid hormones (cortisone, cortisol, androstenedione, testosterone, and progesterone), ^13^C-labeled analogs were used as surrogate analytes, and for the eCBs (AEA, 1-AG/2-AG, OEA, and PEA), deuterated analogs were used. Figure 1 shows a chromatogram of the surrogate analytes and Table S1 in Supplemental summarizes each authentic analyte, its surrogate analyte, and the internal standard. Good separation could be achieved for every analyte in a total run time of 16 min.

### Extraction efficiency

**Fig. 2:**
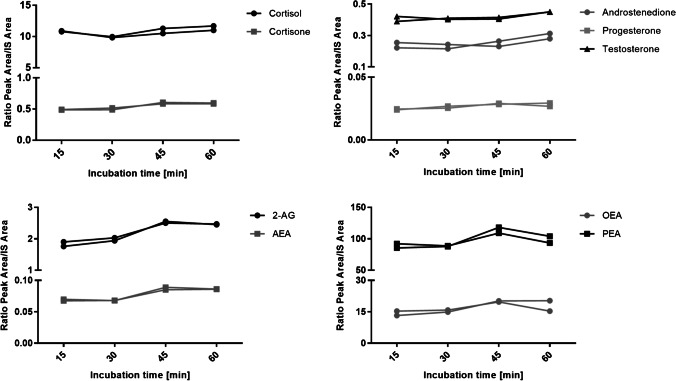
Extraction efficiency from an authentic nail pool (ratio peak area/IS area) depending on different incubation times (*N* = 2)

We first tested the extraction efficiency from the authentic nail pools. A previously published method for extraction of eCBs from hair was used [22]. In brief, methanolic extraction was performed in an ultrasonic bath at 55 °C for 4 h. Additionally, different solvents (methanol, acetone, toluene, and the solvent mixture acetone/methanol 1/5 v/v as well as various methanol/toluene mixtures, 10/1 v/v, 5/1 v/v, and 3/1 v/v) were tested for extraction but no improvement compared to methanolic extraction could be achieved (data not shown). Furthermore, different extraction times were evaluated and after extraction for 1 h, saturation could be reached for all the analytes (Fig. 2). In conclusion, the extraction time could be shortened to 1 h compared to the protocol for hair extraction.

### Validation

The validation was performed in accordance with the guidelines of the Society of Toxicological and Forensic Chemistry (GTFCh) for validation in hair [24]. The validation was carried out with the help of surrogate analytes (D_4_-AEA, D_5_-2-AG, D_4_-OEA, D_4_-PEA, ^13^C_3_-cortisol, ^13^C_3_-cortisone, ^13^C_3_-androstenedione, ^13^C_3_-progesterone, and ^13^C_3_-testosterone). The response factor was determined for every surrogate and authentic analyte. The results are shown in Table S4 in Supplemental. The ideal theoretical response factor would be 1. All analytes showed a variation from the labeled to the authentic analytes. Therefore, the regression line of the calibration curve was corrected by the response factor. OEA showed the biggest variation from the theoretical value of 1. The selectivity of the method could be proven for all analytes. Unspiked nail samples were measured, and no interferences at the retention time of the surrogate analytes could be detected. Furthermore, selectivity was tested in neat solution for surrogate and authentic analytes to confirm that no false-positive signal would appear. The selectivity of D_4_-AEA in a neat solution is exemplified in Fig S1 in Supplemental.

A summary of the calibration curves and LOQ is provided in supplemental Table S5. Linearity was good for all analytes with a correlation coefficient (*R*^2^) of more than 0.98. The equation for the calibration curve was quadratic for the analytes D_4_-OEA and D_4_-PEA and linear for the other analytes. Matrix effects (ion enhancement or ion suppression) were observed for some analytes but were still in the acceptable range of 70–130% for all analytes (Figure S2 in Supplemental and Table S6) except for D_5_-2-AG, which showed an ion enhancement of 67 and 65%. The results for accuracy and precision are summarized in Table S6 in Supplemental. The accuracy for all analytes was in an acceptable range of ± 20%. The precision was in the range of ± 20% for these analytes. For robustness, six replicates of a homogenous and authentic nail pool were measured. The mean and the relative standard deviation were calculated and are displayed in Table S7. The relative standard deviations ranged from 0.5 to 20.2% which is in an acceptable range. The same pool was analyzed with every measured batch for validation and authentic pools during this whole project which was conducted within a time frame of over 6 months. The stability of the pool was monitored over this time frame and was shown to be stable (deviation below 20%). The stability of the processed samples (QCs and an authentic pool) in the autosampler (at 4 °C) was monitored after 24 h, representing a standard batch (see Table S13). No significant instability was observed for 24 h for any of the analytes (data shown in Table S13). All analytes were stable in the authentic pool over the period of the batch analysis.

### Effect of nail weight

**Fig. 3:**
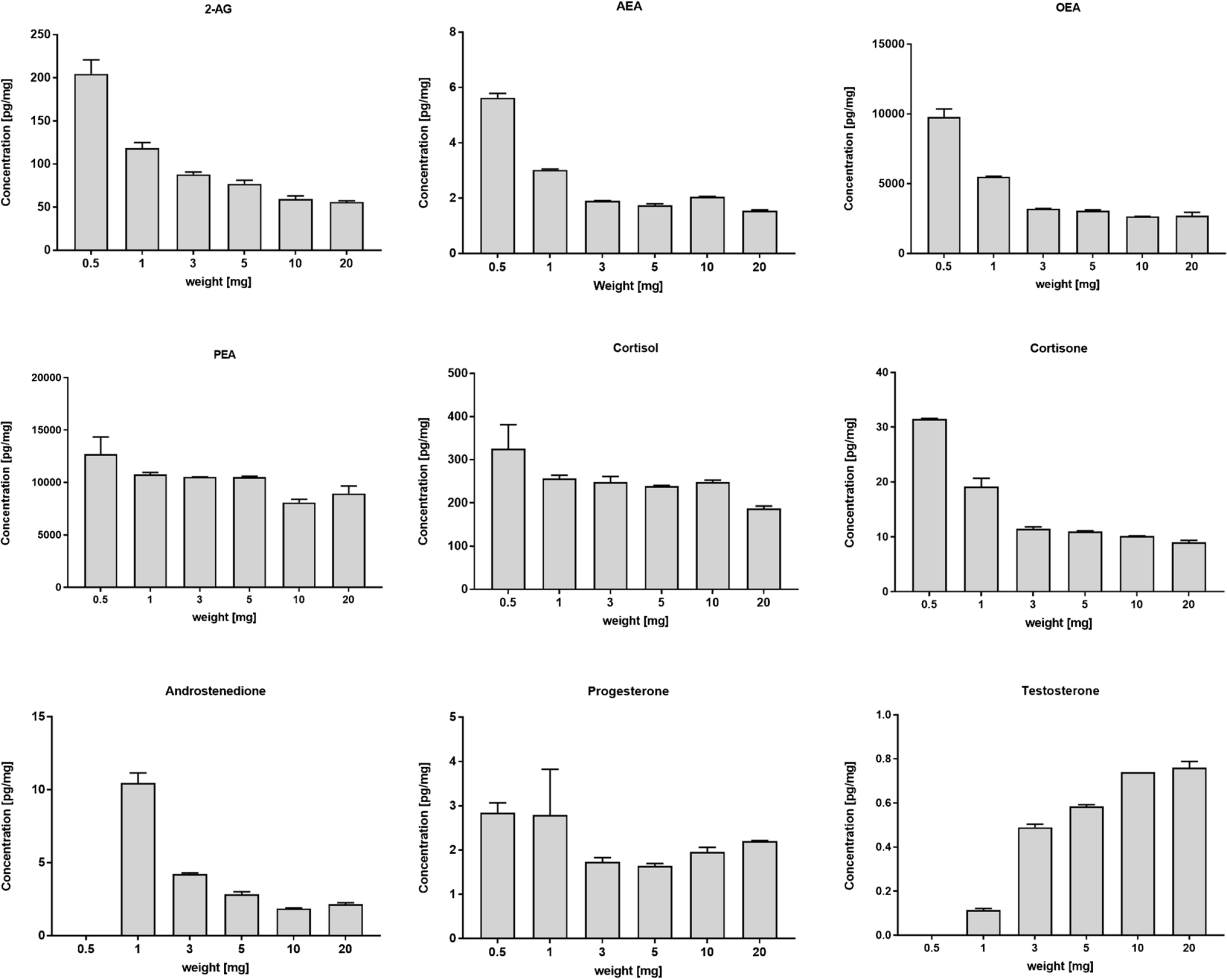
Concentration of endogenous analytes in homogenized nail pools at different sample weights. The error bars at the end of the bars represent the standard deviation (*N* = 2)

The effect of nail weight on the steroid and eCB concentrations was tested by analyzing different amounts of sample weight from homogenized nail pools. Five different pools were extracted with increasing sample weight (0.5, 1, 3, 5, 10 and 20 mg; data shown in Tables S8–S12). In a representative sample, analyte concentrations were stable for most of the substances with 5 mg or higher sample weight (Fig. 3). Lower sample weight mostly resulted in higher analyte concentrations.

### Proof of concept: measurement of authentic nail samples

The newly adapted and validated analytical method was applied to authentic nail samples to analyze steroid and eCB concentrations in the nail matrix of healthy volunteers. Therefore, 57 authentic nail samples (37 nail samples of 12 children, age range: 1 month–8 years; 20 nail samples of 4 mothers and 2 fathers, age range: 32–43 years; for details, see Table S2) were analyzed. Table 1 shows the mean and median concentrations of all samples.

**Table 1:** Mean and median (range) analyte concentrations in nails of children and parents. *N* refers to the number of samples

Analytes	Children	Mothers	Fathers
	Mean; median (range) [pg/mg]	Mean; median (range) [pg/mg]	Mean; median (range) [pg/mg]
2-AG	39.4; 32.5 (16.9–164) *N* = 36	25.8; 29.7 (7.7–41.3) *N* = 16	24.4; 24.5 (13.6–34.9) *N* = 4
AEA	1.8; 1.7 (0.9–4.5) *N* = 32	0.7; 0.7 (0.5–1.0) *N* = 16	1.2; 1.1 (0.6–2.2) *N* = 4
OEA	922; 835 (346–2163) *N* = 37	892; 885 (206–1565) *N* = 16	1197; 909 (653–2317) *N* = 4
PEA	2747; 2159 (728–6694) *N* = 37	3232; 3767 (679–5833) *N* = 16	2714; 2153 (1030–5520) *N* = 4
Cortisol	1.4; 1.3 (0.4–4.2) *N* = 24	1.0; 1.0 (0.4–1.9) *N* = 15	1.9; 1.6 (0.4–3.8) *N* = 4
Cortisone	13.8; 5.6 (1.5–143) *N* = 37	2.9; 2.8 (1.5–4.3) *N* = 15	4.1; 3.7 (1.9–6.9) *N* = 4
Androstenedione	0.9; 0.6 (0.4–2.1) *N* = 14	0.7; 0.7 (0.4–1.3) *N* = 16	0.6; 0.6 (0.4–0.8) *N* = 4
Progesterone	1.8; **1.5** (0.5–8.5) *N* = 33	2.9; 1.3 (0.7–12.8) *N* = 16	1.6; 1.6 (1.0–2.1) *N* = 4
Testosterone	0.4; 0.4 (0.3–0.5) *N* = 2	n.d	0.6; 0.5 (0.4–0.8) *N* = 4

*n.d.* not detectable

**Fig. 4:**
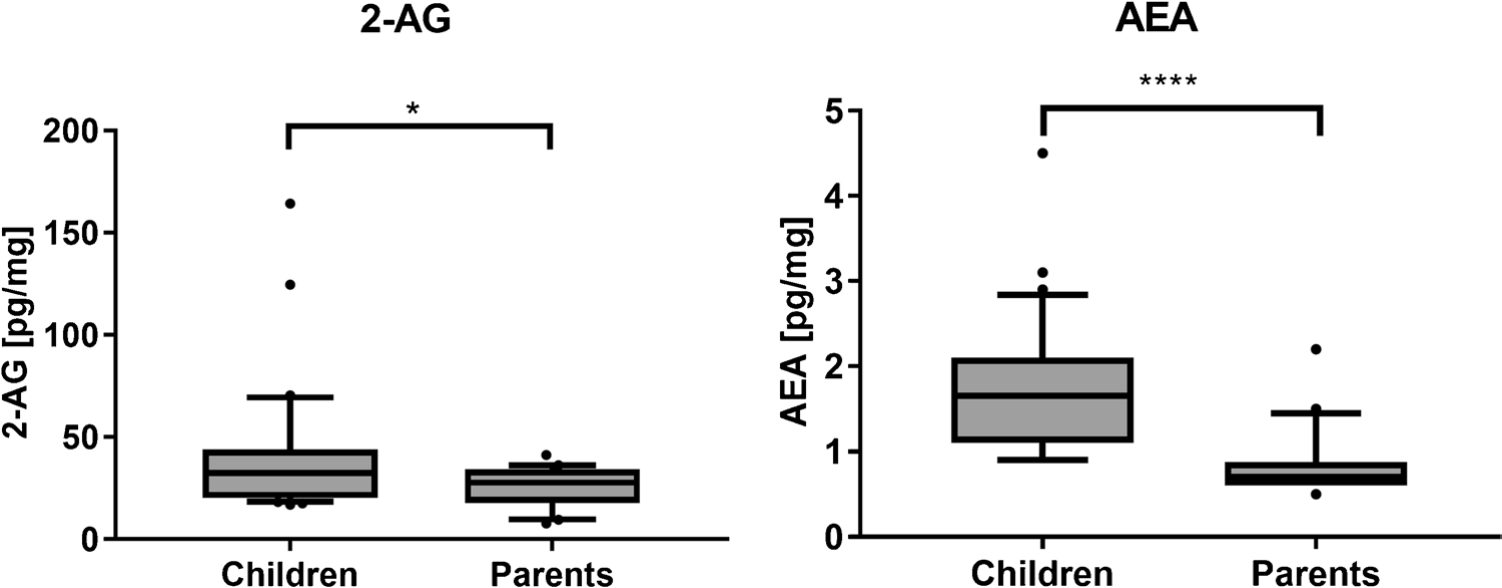
Statistical analysis of eCB levels in children and parents. Box plots with median and interquartile range and 10% and 90% percentiles. The statistical analysis was conducted using the Mann–Whitney test. The significance level is indicated with asterisks

Statistical analysis of eCBs and steroid concentrations in nails of children and parents showed a significant difference for 2-AG and AEA (Mann–Whitney test; Fig. 4). No significant difference could be found for the other analytes.

Correlations for eCBs and steroid hormones were calculated. AEA showed a significant, positive correlation with 2-AG (*r* = 0.52, *p* < 0.0001), OEA was significantly and positively correlated with PEA (*r* = 0.80, *p* < 0.0001), and a positive correlation was found between cortisol and cortisone (*r* = 0.69, *p* < 0.0001) and progesterone and androstenedione (*r* = 0.69, *p* < 0.0001). In addition, a significant correlation was found between AEA and cortisone (*r* = 0.55, *p* < 0.0001). Correlations of eCBs with each other have been found in hair before [22, 27]. These results indicate that the effects seem to be similar in keratinized matrices. For nail analysis, it is important to determine whether there are significant differences of steroid concentrations in the right and left hand. A comparison between substance concentrations in fingernails from the left and right hand of four to eight individuals is shown in Table 2. The analyte concentrations of each hand (left and right) showed no significant differences (*p* > 0.05) except for 2-AG (*p* < 0.05).

**Table 2:** Mean and median (range) analyte concentrations in the left and right hand and Spearman correlation between the left and right hand. *N* = number of pairs

Analytes	Left hand	Right hand	*N*	Spearman *r*
	Mean; median (range) [pg/mg]	Mean; median (range) [pg/mg]		*r* (significance)
2-AG	30.5; 32.4 (18.8–41.3)	27.4; 29.2 (17.5–36.5)	8	0.90 (*p* < 0.01)
AEA	1.3; 1.2 (0.5–2.1)	1.1; 1.1 (0.7–1.9)	8	0.90 (*p* < 0.01)
OEA	1038.0; 1007 (516.5–1890)	1016.0; 924.4 (679.5–1567)	8	0.81 (*p* < 0.05)
PEA	3926.0; 3898 (2159–6694)	4071.0; 3608 (2880–6511)	8	0.76 (*p* < 0.05)
Cortisol	1.0; 1.1 (0.8–1.5)	1.1; 1.1 (0.7–1.4)	4	1.00 (*p* < 0.05)
Cortisone	4.0; 3.6 (2.5–6.6)	4.5; 4.2 (2.7–7.5)	8	0.85 (*p* < 0.05)
Androstenedione	0.7; 0.5 (0.4–1.1)	0.8; 0.8 (0.5–1.3)	6	0.99 (*p* < 0.05)
Progesterone	4.0; 1.2 (0.8–12.8)	2.6; 1.6 (0.9–8.2)	8	0.76 (*p* < 0.05)
Testosterone	n.d	n.d	–	–

*n.d.* not detectable

## Discussion

In recent years, nail cortisol has emerged as a promising biomarker for researching the consequences of chronic stress exposure on HPA axis function [28]. The additional inclusion of eCBs as potential indicators for stress adaptation processes opens up new possibilities for future stress research. This pilot study intended to generate the first empirical values of eCBs in nails and to establish nails as a suitable matrix for the retrospective and simultaneous monitoring of cumulative steroid hormone and eCB levels using a validated and sensitive LC–MS/MS method. In the current study, the sample preparation including methanolic extraction was successfully established for nail samples. The method could be optimized to an incubation time of 1 h in comparison to hair samples [22]. This improvement does not only save time, but also reduces the exposure to plastic and glass surfaces, which may potentially adsorb the target substances [29]. Furthermore, the extraction solvent is very important for efficient extraction. Keratinized matrices, such as nails, require a solvent that both dissolves the analytes sufficiently and simultaneously swells the matrix. After different solvents were tested, methanol was found to be the most efficient extraction reagent. Because extraction from keratinized matrices can also incorporate interfering substances, further clean-up steps are required. Voegel et al. [22] have already shown that supported liquid extraction (SLE) enables an efficient clean-up and recovery of all analytes in the keratinized matrix hair and can decrease background noise. The same approach was now successfully used in nails. The publication by Voegel et al. showed that using surrogate analytes for steroid analytics [22] is an elegant way to quantify endogenous substances when no analyte-free matrix is available. The analytical method described here has been optimized by using surrogate analytes (^13^C-labeled and deuterated) for each authentic analyte, which now enables the accurate quantification of a full panel (steroids and eCBs) of endogenous substances from nails. For the quantification of OEA, it has to be taken into account that vaccenic acid (cis11-18:1) ethanolamide (VEA) has the same mass and the same fragmentation pattern as OEA and was found in rat and human plasma [30]. It cannot be excluded that VEA is present in human nails. The LC–MS/MS method was successfully validated and performed in adaption to the guidelines of the GTFCh for validation in hair [31]. Matrix effects with ion enhancement occurred for 2-AG, OEA, and PEA. Accuracy and precision were in the accepted range of ± 20% for all substances. Robustness and stability experiments using QCs and authentic pools proved that the analytes are stable in the course of sample preparation and allow an adequate quantification.

Systematic studies in the field of nail analysis are still scarce [19, 32]. Our results show that depending on nail weight, the substance concentrations can vary. Nevertheless, for some analytes (cortisol, cortisone, 2-AG, AEA and PEA), the substance concentrations were very stable. We suggest that the minimum amount of nail samples for a reliable detection of all analytes should be 5 mg, as it seems that for very small amounts (< 1 mg) the variation becomes more significant. For some analytes (e.g., progesterone), even 0.5 to 3 mg produced reliable data. Interestingly, some analytes showed higher concentrations when small sample amounts were used, which could also be interpreted as a matrix effect. Our study is the first to systematically investigate the effect of nail weight on the analyte concentration. Even if the reason for the observed effect is still unclear, the results show that a relatively small amount (5 mg) of nails can yield stable results. This is important in newborn research, where only tiny nail samples are available. Further research using small sample amounts is needed in order to interpret this result. In this proof-of-concept study, 57 samples were measured and evaluated. To the best of our knowledge, this is the first time that eCBs were successfully detected in child and adult nails and these data are the first data that are received in this field of research. Of note, testosterone could not be detected in a number of samples, mostly in those from women and children. Testosterone is a male sex hormone [33], and concentrations in women and children are known to be very low. Thus, the limitations in testosterone measurements in women and children can be most certainly ascribed to limited sensitivity at the concentration range in these samples [34]. As presented in Table 1 and Fig. 2, there is a significant concentration difference between children’s and parents’ nail concentrations for 2-AG and AEA. For the other substances, there were no significant differences. Whether this difference is correlated to age can only be speculated, and further analysis has to be done to investigate age effects on stress marker concentrations in nails. In any case, it is important to consider age-dependent eCB levels in nails, similar to what has been suggested for hair [22, 35, 36]. As can be seen in Table S2, the children’s nail samples did not always have a weight of 5 mg, and one infant nail sample (mixture of left and right hands) weighed only about 2.5 mg. This is an important observation for future studies, as our findings show that in small infants it is necessary to collect repeated nail clippings to have enough sample material for a robust measurement. There were no significant concentration differences between the left and right hand for most of the substances. Similar results have been found previously for testosterone and cortisol in nails [16, 35], indicating that sample collection does not need to be limited to one hand alone, providing an advantage for sample collection. The relative ratio of measured analyte concentrations in nails (data not shown) was in good accordance with concentrations published previously in hair [22]. Yet, compared to hair analyses, the concentrations of endogenous analytes in nails are lower. This is also in accordance with other findings [23, 26]. The difference in analyte concentrations in nail and hair samples might be explained by several factors, such as melanin binding, physicochemical properties of the incorporated substances, incorporation mechanisms, and surfaces of the two different matrices. Furthermore, hair grows with a growth rate of roughly 1 cm/month [37] which is about two to three times faster than fingernails with 0.4 cm/month [38, 39]. Three routes for substance incorporation into the nail matrix have been suggested: with the primary mechanism through the germinal nail matrix, followed by incorporation from the nail bed and contamination from sweat [40, 41]. Thus, due to the continuous nail matrix keratinization, substances are distributed over the entire nail as summarized by Baswan et al. [42]. This study has several strengths: Due to the surrogate analytes, the quantity of both steroid hormones and eCBs could be accurately determined in nails. The findings were reliable even though only small amounts of nail matrix were available. It is easy to both collect and store nails in a reliable and reproducible manner. We were able to involve samples of both men and women as well as children.

## Limitations

However, there are also limitations of the study. Due to the lack of large reliable studies including nail analyses, the findings are difficult to compare and normative values still need to be generated. Furthermore, quantification of endogenous compounds like eCBs is analytically challenging. In our opinion, the approach of the surrogate analyte quantification is the best approach so far, but the lack of internal standards for each analyte is still a drawback. Furthermore, the influence of detergents, hand cream, or nail polish on hormone analysis in nails is still unclear and needs further investigation. Our study only included a limited number of samples, and the nail weights of some newborn samples were below 5 mg. The recommended sample weight from our study to get reliable results is 5 mg. We are aware of the fact that this might still be a high amount when investigating newborn fingernails. Nevertheless, this is the first time that nine different stress markers could be detected from one sample, which constitutes a major improvement for stress analysis. One suggestion to obtain enough sample weight would therefore be to collect the first 5–6 nail cuts of the newborns. These nail cuts could be subsequently pooled and analyzed. As mentioned above, analyte concentrations can vary if the sample amount is too small. For a better conclusion, further studies with larger sample sizes are needed. Also, the establishment of normative or cut-off values would be highly beneficial. However, data suggest that nail matrix analysis might be a promising substitute for other keratinized matrices [43]. The correlation of nail concentrations to hair concentrations would be very interesting, but in our individuals, there was either no hair available (newborns, some males) or mainly cosmetically treated (women).

## Conclusion

A sensitive LC–MS/MS method was successfully established and validated for the simultaneous quantification of four eCBs and five steroid hormones in human fingernail clippings. This is the first time that eCBs could be determined and quantified in nails. The combined assessment of steroids and eCB concentrations from fingernail samples can be used for the retrospective measurement of these substances in adults and children and might be a promising new research tool for gaining further important insights into the psychophysiological mechanisms of stress.

## Supplementary Information

Below is the link to the electronic supplementary material.

Supplementary file1 (DOCX 383 KB)

## Abbreviations

ACTH: Adrenocorticotropic hormone
AEA: Anandamide
2-AG: Arachidonoylglycerol
CRH: Corticotropin-releasing hormone
CB: Cannabinoid
eCB: Endocannabinoid
HPA: Hypothalamic–pituitary–adrenal axis
LC–MS/MS: Liquid chromatography–tandem mass spectrometry
LOQ: Limit of quantification
MeOH: Methanol
OEA: Oleoylethanolamide
PEA: Palmitoylethanolamide
QC: Quality control
SLE: Supported liquid extraction

## Declarations

### Source of biological material

Nail samples were obtained from volunteers after oral informed consent. For children, the informed consent of the parents was obtained.

## References

[1] McEwen BS (1998). Protective and damaging effects of stress mediators. N Engl J Med.

[2] Russell G, Lightman S (2019). The human stress response. Nat Rev Endocrinol.

[3] Hellhammer DH, Wust S, Kudielka BM (2009). Salivary cortisol as a biomarker in stress research. Psychoneuroendocrinology.

[4] Riebe CJ, Wotjak CT (2011). Endocannabinoids and stress. Stress.

[5] Zanettini C, Panlilio LV, Alicki M, Goldberg SR, Haller J, Yasar S (2011). Effects of endocannabinoid system modulation on cognitive and emotional behavior. Front Behav Neurosci.

[6] Devane WA, Hanus L, Breuer A, Pertwee RG, Stevenson LA, Griffin G (1992). Isolation and structure of a brain constituent that binds to the cannabinoid receptor. Science.

[7] Herkenham M, Lynn AB, Little MD, Johnson MR, Melvin LS, de Costa BR (1990). Cannabinoid receptor localization in brain. Proc Natl Acad Sci U S A.

[8] Lutz B, Marsicano G, Maldonado R, Hillard CJ (2015). The endocannabinoid system in guarding against fear, anxiety and stress. Nat Rev Neurosci.

[9] Ho WS, Barrett DA, Randall MD (2008). ‛Entourage’ effects of N-palmitoylethanolamide and N-oleoylethanolamide on vasorelaxation to anandamide occur through TRPV1 receptors. Br J Pharmacol.

[10] Hill MN, Kumar SA, Filipski SB, Iverson M, Stuhr KL, Keith JM (2013). Disruption of fatty acid amide hydrolase activity prevents the effects of chronic stress on anxiety and amygdalar microstructure. Mol Psychiatry.

[11] Mayo LM, Asratian A, Lindé J, Morena M, Haataja R, Hammar V (2020). Elevated anandamide, enhanced recall of fear extinction, and attenuated stress responses following inhibition of fatty acid amide hydrolase: a randomized, controlled experimental medicine trial. Biol Psychiatry.

[12] Lewis JG (2006). Steroid analysis in saliva: an overview. Clin Biochem Rev.

[13] Pruessner JC, Wolf OT, Hellhammer DH, Buske-Kirschbaum A, von Auer K, Jobst S (1997). Free cortisol levels after awakening: a reliable biological marker for the assessment of adrenocortical activity. Life Sci.

[14] Ostheim P, Tichý A, Sirak I, Davidkova M, Stastna MM, Kultova G (2020). Overcoming challenges in human saliva gene expression measurements. Sci Rep.

[15] Thongboonkerd V, Saetun P (2007). Bacterial overgrowth affects urinary proteome analysis: recommendation for centrifugation, temperature, duration, and the use of preservatives during sample collection. J Proteome Res.

[16] Voegel CD, La Marca-Ghaemmaghami P, Ehlert U, Baumgartner MR, Kraemer T, Binz TM (2018). Steroid profiling in nails using liquid chromatography-tandem mass spectrometry. Steroids.

[17] Stalder T, Steudte-Schmiedgen S, Alexander N, Klucken T, Vater A, Wichmann S (2017). Stress-related and basic determinants of hair cortisol in humans: a meta-analysis. Psychoneuroendocrinology.

[18] Tegethoff M, Raul JS, Jamey C, Khelil MB, Ludes B, Meinlschmidt G (2011). Dehydroepiandrosterone in nails of infants: a potential biomarker of intrauterine responses to maternal stress. Biol Psychol.

[19] Fischer S, Schumacher S, Skoluda N, Strahler J (2020). Fingernail cortisol - state of research and future directions. Front Neuroendocrinol.

[20] Stalder T, Kirschbaum C (2012). Analysis of cortisol in hair–state of the art and future directions. Brain Behav Immun.

[21] van de Merbel NC (2008). Quantitative determination of endogenous compounds in biological samples using chromatographic techniques. TrAC, Trends Anal Chem.

[22] Voegel CD, Baumgartner MR, Kraemer T, Wust S, Binz TM (2021). Simultaneous quantification of steroid hormones and endocannabinoids (ECs) in human hair using an automated supported liquid extraction (SLE) and LC-MS/MS - insights into EC baseline values and correlation to steroid concentrations. Talanta.

[23] Voegel CD, Hofmann M, Kraemer T, Baumgartner MR, Binz TM (2020). Endogenous steroid hormones in hair: investigations on different hair types, pigmentation effects and correlation to nails. Steroids.

[24] Mußhoff F, Skopp G, Pragst F, Sachs H, Thieme D (2009). Appendix C to the GTFCh guidelines for quality assurance inforensic-toxicological analyses. Toxichem Krimtech.

[25] Binz TM, Braun U, Baumgartner MR, Kraemer T (2016). Development of an LC-MS/MS method for the determination of endogenous cortisol in hair using (13)C3-labeled cortisol as surrogate analyte. J Chromatogr B Analyt Technol Biomed Life Sci.

[26] Binz TM, Gaehler F, Voegel CD, Hofmann M, Baumgartner MR, Kraemer T (2018). Systematic investigations of endogenous cortisol and cortisone in nails by LC-MS/MS and correlation to hair. Anal Bioanal Chem.

[27] Gao W, Walther A, Wekenborg M, Penz M, Kirschbaum C (2020). Determination of endocannabinoids and N-acylethanolamines in human hair with LC-MS/MS and their relation to symptoms of depression, burnout, and anxiety. Talanta.

[28] Liu CH, Doan SN (2019). Innovations in biological assessments of chronic stress through hair and nail cortisol: conceptual, developmental, and methodological issues. Dev Psychobiol.

[29] Karlsson M, Påhlsson C, Fowler CJ (2004). Reversible, temperature-dependent, and AM404-inhibitable adsorption of anandamide to cell culture wells as a confounding factor in release experiments. Eur J Pharm Sci.

[30] Röhrig W, Waibel R, Perlwitz C, Pischetsrieder M, Hoch T (2016). Identification of the oleic acid ethanolamide (OEA) isomer cis-vaccenic acid ethanolamide (VEA) as a highly abundant 18:1 fatty acid ethanolamide in blood plasma from rats and humans. Anal Bioanal Chem.

[31] Musshoff F, Skopp G, Pragst FSH. Appendix C of the GTFCh guidelines for quality control in forensic-toxicological analyses. Quality Requirements for the Analysis of Hair Samples. 2009.

[32] Phillips R, Kraeuter AK, McDermott B, Lupien S, Sarnyai Z (2021). Human nail cortisol as a retrospective biomarker of chronic stress: a systematic review. Psychoneuroendocrinology.

[33] Yildirim BO, Derksen JJL (2012). A review on the relationship between testosterone and life-course persistent antisocial behavior. Psychiatry Res.

[34] Rosner W, Auchus RJ, Azziz R, Sluss PM, Raff H (2007). Utility, limitations, and pitfalls in measuring testosterone: an Endocrine Society position statement. J Clin Endocrinol Metab.

[35] Lanfear JH, Voegel CD, Binz TM, Paul RA (2020). Hair cortisol measurement in older adults: Influence of demographic and physiological factors and correlation with perceived stress. Steroids.

[36] Binz TM, Rietschel L, Streit F, Hofmann M, Gehrke J, Herdener M (2018). Endogenous cortisol in keratinized matrices: systematic determination of baseline cortisol levels in hair and the influence of sex, age and hair color. Forensic Sci Int.

[37] Xiang L, Sunesara I, Rehm KE, Marshall GD (2016). Hair cortisol concentrations are associated with hair growth rate. NeuroImmunoModulation.

[38] Altan Ferhatoglu Z, Goktay F, Yasar S, Aytekin S (2018). Morphology, growth rate, and thickness of the nail plate during the pregnancy. Int J Dermatol.

[39] Yaemsiri S, Hou N, Slining MM, He K (2010). Growth rate of human fingernails and toenails in healthy American young adults. J Eur Acad Dermatol Venereol : JEADV.

[40] Solimini R, Minutillo A, Kyriakou C, Pichini S, Pacifici R, Busardo FP (2017). Nails in forensic toxicology: an update. Curr Pharm Des.

[41] Madry MM, Steuer AE, Binz TM, Baumgartner MR, Kraemer T (2014). Systematic investigation of the incorporation mechanisms of zolpidem in fingernails. Drug Test Anal.

[42] Baswan S, Kasting GB, Li SK, Wickett R, Adams B, Eurich S (2017). Understanding the formidable nail barrier: a review of the nail microstructure, composition and diseases. Mycoses.

[43] Krumbiegel F, Hastedt M, Tsokos M (2014). Nails are a potential alternative matrix to hair for drug analysis in general unknown screenings by liquid-chromatography quadrupole time-of-flight mass spectrometry. Forensic Sci Med Pathol.

