# Supplementary material for: Endocannabinoid and steroid analysis in infant and adult nails by LC–MS/MS
Source: Anal Bioanal Chem. 2022 Jul 4;414(20):6201–11. doi: 10.1007/s00216-022-04189-y (PMC9314307; doi:10.1007/s00216-022-04189-y)
Supplement: Supplementary file 1 — Supplementary file1 (DOCX 383 KB) [file 216_2022_4189_MOESM1_ESM.docx]

**Supplemental**

**Tables**

Table S1: All analytes listed with their corresponding internal standard and the surrogate analyte used for the calibration.

| **Analyte** | **Internal standard** | **Analyte for calibration** |
| --- | --- | --- |
| 2-AG | D_11_-AEA | D_5_-2-AG |
| AEA | D_11_-AEA | D_4_-AEA |
| Androstenedione | D_9_-progesterone | ^13^C_3_-Androstenedione |
| Cortisol | D_7_-cortisone | ^13^C_3_-Cortisol |
| Cortisone | D_7_-cortisone | ^13^C_3_-Cortisone |
| OEA | D_11_-AEA | D_4_-OEA |
| PEA | D_11_-AEA | D_4_-PEA |
| Progesterone | D_9_-progesterone | ^13^C_3_-Progesterone |
| Testosterone | D_9_-progesterone | ^13^C_3_-Testosterone |

Table S2: Measurement results of authentic samples: N=57 samples, n.d.: not detected

| **Sample** | **Age** | **Amount** | **2-AG** | **AEA** | **OEA** | **PEA** | **Cortisol** | **Cortisone** | **Androstenedione** | **Progesterone** | **Testosterone** |
| --- | --- | --- | --- | --- | --- | --- | --- | --- | --- | --- | --- |
|  |  | [mg] | [pg/mg] | [pg/mg] | [pg/mg] | [pg/mg] | [pg/mg] | [pg/mg] | [pg/mg] | [pg/mg] | [pg/mg] |
| K1_LR | 1 month | 2.6 | 70.4 | 2.7 | 685.8 | 1145.4 | 2.7 | 20.4 | 2.1 | 4.6 | n.d. |
| K10 | 1 year | 0.9 | 124.6 | 1.9 | 1068.2 | 1259.1 | n.d. | 3.5 | n.d. | 1.8 | n.d. |
| K2_F1 | 5 years | 20.8 | 35.4 | 1.7 | 525.6 | 798.0 | 0.9 | 4.6 | 0.1 | 0.5 | n.d. |
| K2_F2 | 5 years | 19.9 | 19.8 | 1.2 | 426.9 | 998.5 | 0.6 | 3.8 | 0.0 | 0.5 | n.d. |
| K2_F3 | 5 years | 24.6 | 23.4 | 1.0 | 567.0 | 1100.2 | 1.1 | 5.4 | 0.0 | 0.5 | n.d. |
| K2_H1 | 5 years | 21.0 | 57.0 | 2.5 | 1784.2 | 4446.0 | 2.2 | 8.2 | 0.1 | 1.0 | n.d. |
| K2_H2 | 5 years | 20.5 | 33.0 | 2.1 | 2162.9 | 6478.9 | 1.6 | 6.5 | 0.0 | 0.8 | n.d. |
| K2_H3 | 5 years | 16.2 | 23.3 | 1.6 | 1565.6 | 4230.1 | 1.6 | 5.7 | 0.0 | 0.8 | n.d. |
| K2_L1 | 5 years | 5.4 | 35.7 | 1.9 | 1087.3 | 4146.3 | 0.2 | 4.8 | 0.6 | 5.7 | n.d. |
| K2_L2 | 5 years | 8.7 | 21.5 | 2.1 | 1132.7 | 3797.6 | 0.5 | 5.4 | 0.4 | 1.0 | n.d. |
| K2_L3 | 5 years | 6.2 | 25.1 | 1.8 | 1889.6 | 6693.8 | 0.0 | 6.6 | 0.5 | 8.5 | n.d. |
| K2_R1 | 5 years | 6.0 | 28.8 | 1.9 | 1412.6 | 5715.3 | 0.1 | 5.0 | 0.6 | 2.7 | n.d. |
| K2_R2 | 5 years | 11.8 | 19.3 | 1.3 | 835.0 | 3075.3 | 1.0 | 6.4 | n.d. | 1.5 | n.d. |
| K2_R3 | 5 years | 8.6 | 19.9 | 1.3 | 1566.7 | 6511.3 | 2.0 | 7.5 | 0.5 | 3.3 | n.d. |
| K3_F1 | 8 years | 19.6 | 18.2 | 0.9 | 353.9 | 1083.9 | 1.1 | 3.6 | 0.0 | 0.4 | n.d. |
| K3_F2 | 8 years | 19.6 | 17.5 | 1.1 | 456.6 | 1224.1 | 1.2 | 3.8 | 0.0 | 0.3 | n.d. |
| K3_H1 | 8 years | 21.0 | 31.9 | 1.8 | 1264.0 | 4452.2 | 1.5 | 5.9 | 0.0 | 0.6 | n.d. |
| K3_H2 | 8 years | 20.3 | 49.0 | 1.6 | 1668.8 | 5998.8 | 1.3 | 5.6 | 0.1 | 0.7 | n.d. |
| K3_L | 5 years | 11.2 | 36.8 | 1.4 | 516.5 | 2158.8 | 0.0 | 2.9 | n.d. | 0.8 | n.d. |
| K3_R | 5 years | 14.7 | 36.5 | 1.3 | 679.5 | 2890.3 | 0.7 | 4.7 | 0.1 | 0.9 | n.d. |
| K4 | unknown | 14.0 | 28.7 | 1.9 | 555.1 | 1563.0 | 1.4 | 9.1 | 0.4 | 2.1 | n.d. |
| K5 A | 6 years | 20.5 | 18.5 | 1.1 | 1013.1 | 4492.0 | 1.2 | 3.6 | 0.2 | 0.5 | n.d. |
| K5 B | 6 years | 20.3 | 16.9 | 1.1 | 854.4 | 3534.7 | 0.9 | 2.9 | 0.2 | 0.4 | n.d. |
| K6 T1 | 11 months | 2.8 | 44.2 | 1.1 | 498.7 | 4540.2 | n.d. | 3.4 | n.d. | 2.4 | n.d. |
| K6 T2 | 11 months | 0.6 | 164.3 | 2.5 | 1569.2 | 2672.5 | n.d. | 6.3 | n.d. | 1.9 | n.d. |
| K6 T3 | 11 months | 1.8 | n.d. | 0.9 | 523.0 | 728.4 | n.d. | 3.8 | n.d. | 1.5 | n.d. |
| K7 T1 | 1 year | 3.1 | 35.1 | n.d. | 481.3 | 1535.6 | n.d. | 7.2 | 1.3 | 2.4 | n.d. |
| K7 T2 | 1 year | 2.0 | 55.1 | n.d. | 601.0 | 1329.5 | n.d. | 9.4 | 1.7 | 3.1 | n.d. |
| K7 T3 | 1 year | 2.5 | 44.1 | n.d. | 458.9 | 868.5 | n.d. | 5.2 | n.d. | 2.1 | n.d. |
| K7 T4 | 1 year | 3.1 | 36.7 | n.d. | 397.3 | 864.0 | n.d. | 6.8 | 1.4 | 1.6 | n.d. |
| K7 T5 | 1 year | 4.4 | 24.9 | n.d. | 345.5 | 762.0 | 0.3 | 9.1 | 0.8 | 1.1 | n.d. |
| K8 | 2 years | 18.9 | 69.0 | 2.6 | 1142.9 | 2595.7 | 1.7 | 7.6 | 0.0 | 0.4 | n.d. |
| K9 | 5 months | 7.2 | 28.6 | 4.5 | 575.2 | 2353.6 | 4.2 | 28.5 | 1.2 | 0.9 | n.d. |
| M1_L | Adult unknown | 20.9 | 41.3 | 1.0 | 1423.3 | 4410.9 | 1.9 | 4.3 | 1.1 | 12.8 | 0.2 |
| M1_R | Adult unknown | 22.6 | 34.4 | 0.8 | 985.7 | 2833.6 | 1.0 | 2.8 | 1.3 | 8.2 | 0.1 |
| M2_F1 | 38 years | 19.8 | 7.7 | 0.6 | 206.0 | 678.9 | 0.2 | 0.8 | 0.6 | 0.9 | 0.0 |
| M2_F2 | 38 years | 23.1 | 9.5 | 0.6 | 388.2 | 1050.8 | 0.4 | 1.5 | 0.7 | 0.9 | 0.1 |
| M2_H1 | 38 years | 21.1 | 32.7 | 0.9 | 1438.8 | 5833.0 | 1.2 | 3.3 | 0.4 | 0.7 | 0.0 |
| M2_H2 | 38 years | 20.0 | 12.5 | 0.7 | 273.2 | 939.3 | 0.7 | 1.9 | 0.5 | 0.8 | n.d. |
| M2_L1 | 38 years | 24.8 | 29.7 | 0.7 | 926.7 | 4063.8 | 1.1 | 2.6 | 0.7 | 1.1 | 0.1 |
| M2_L2 | 38 years | 19.0 | 18.8 | 0.6 | 581.3 | 2740.3 | 0.6 | 2.6 | 0.7 | 0.9 | 0.1 |
| M2_L3 | 38 years | 23.0 | 35.1 | 0.5 | 747.9 | 3998.6 | 1.0 | 2.5 | 0.6 | 1.3 | 0.0 |
| M2_R1 | 38 years | 22.4 | 29.6 | 0.7 | 953.6 | 3534.7 | 1.3 | 3.3 | 0.9 | 1.3 | 0.1 |
| M2_R2 | 38 years | 18.8 | 17.5 | 0.7 | 803.2 | 4059.8 | 1.1 | 3.6 | 1.0 | 1.6 | 0.1 |
| M2_R3 | 38 years | 23.8 | 33.5 | 0.7 | 895.2 | 4280.7 | 0.9 | 2.7 | 0.6 | 1.0 | 0.0 |
| M3 A | 43 years | 20.6 | 36.2 | 0.7 | 1545.0 | 4372.2 | 1.0 | 3.5 | 0.6 | 1.5 | 0.1 |
| M3 B | 43 years | 20.3 | 30.0 | 0.9 | 1564.7 | 4055.1 | 1.0 | 4.0 | 0.5 | 1.4 | 0.1 |
| P21-607 A | Pool 3/2 years | 20.0 | 43.3 | 3.1 | 816.0 | 1146.2 | 0.5 | 1.7 | 0.1 | 0.6 | 0.0 |
| P21-607 B | Pool 3/2 years | 20.1 | 43.2 | 2.9 | 867.4 | 1244.5 | 0.4 | 1.5 | 0.1 | 0.6 | 0.3 |
| P21-608 A | Pool 1 year | 20.0 | 18.6 | 0.9 | 865.9 | 1620.5 | 2.1 | 141.7 | 0.4 | 1.6 | 0.5 |
| P21-608 B | Pool 1 year | 19.9 | 18.9 | 0.9 | 886.0 | 1582.2 | 2.1 | 143.1 | 0.5 | 1.6 | 0.1 |
| P21-610 A | Adult unknown | 20.2 | 19.6 | 0.7 | 663.9 | 2114.5 | 0.5 | 2.1 | 0.8 | 5.2 | 0.1 |
| P21-610 B | Adult unknown | 20.0 | 24.1 | 0.6 | 874.1 | 2740.7 | 0.8 | 3.4 | 0.9 | 6.0 | 0.2 |
| V1 A | 43 years | 20.8 | 23.1 | 0.6 | 652.9 | 1299.5 | 0.4 | 2.7 | 0.7 | 1.3 | 0.4 |
| V1 B | 43 years | 20.3 | 13.6 | 0.6 | 663.3 | 1030.2 | 0.4 | 1.9 | 0.5 | 1.0 | 0.1 |
| V2 A | 32 years | 19.8 | 25.8 | 1.5 | 1153.9 | 3005.8 | 2.8 | 4.7 | 0.4 | 2.1 | 0.5 |
| V2 B | 32 years | 20.7 | 34.9 | 2.2 | 2317.3 | 5520.2 | 3.8 | 6.9 | 0.8 | 1.8 | 0.8 |

Table S3: MRM transitions and MS parameters for all analytes and the internal standards. For each substance, the most sensitive transition was used for quantitation (quantifier) and the second sensitive was used for confirmation (qualifier). RT = retention time, DP = declustering potential, EP = entrance potential, CE = collision energy, CXP = cell exit potential.

| **Compound** | **Q1 mass**  **[Da]** | **Q3 mass**  **[Da]** | **RT**  **[min]** | **DP**  **[V]** | **EP**  **[V]** | **CE**  **[V]** | **CXP**  **[V]** |
| --- | --- | --- | --- | --- | --- | --- | --- |
| 2-AG 1 | 379.035 | 287.1 | 10.20 | 21 | 10 | 17 | 14 |
| 2-AG 2 | 379.035 | 269.1 | 10.20 | 21 | 10 | 13 | 12 |
| 2-AG D_5_ 1 | 384.324 | 287.2 | 10.20 | 71 | 10 | 21 | 16 |
| 2-AG D_5_ 2 | 384.324 | 90.9 | 10.20 | 71 | 10 | 71 | 14 |
| AEA 1 | 348.193 | 287.1 | 9.70 | 26 | 10 | 13 | 10 |
| AEA 2 | 348.193 | 61.9 | 9.70 | 26 | 10 | 13 | 10 |
| AEA D_4_ 1 | 352.352 | 66.2 | 9.68 | 56 | 10 | 17 | 10 |
| AEA D_4_ 2 | 352.352 | 91.0 | 9.68 | 56 | 10 | 61 | 10 |
| AEA D_11_ | 359.265 | 61.9 | 9.63 | 1 | 10 | 13 | 8 |
| Androstenedione 1 | 286.984 | 97.0 | 4.77 | 145 | 10 | 46 | 11 |
| Androstenedione 2 | 286.984 | 109.2 | 4.77 | 145 | 10 | 46 | 11 |
| Androstenedione ^13^C_3_ 1 | 290.123 | 100.2 | 4.76 | 61 | 10 | 31 | 16 |
| Androstenedione ^13^C_3_ 2 | 290.123 | 112.1 | 4.76 | 145 | 10 | 46 | 11 |
| N-OEA 1 | 326.160 | 62.1 | 10.85 | 150 | 10 | 13 | 6 |
| N-OEA 2 | 326.160 | 265.3 | 10.85 | 150 | 10 | 13 | 6 |
| N-OEA D_4_ 1 | 330.305 | 313.4 | 10.82 | 46 | 10 | 21 | 22 |
| N-OEA D_4_ 2 | 330.305 | 66.1 | 10.82 | 46 | 10 | 21 | 8 |
| N-PEA 1 | 301.115 | 63.1 | 10.12 | 36 | 10 | 13 | 16 |
| N-PEA 2 | 300.115 | 109.2 | 10.12 | 36 | 10 | 13 | 16 |
| N-PEA D_4_ 1 | 304.296 | 66.1 | 10.10 | 200 | 10 | 19 | 8 |
| N-PEA D_4_ 2 | 304.296 | 287.3 | 10.10 | 16 | 10 | 21 | 42 |
| Cortisol 1 | 363.049 | 121.0 | 3.16 | 145 | 10 | 46 | 11 |
| Cortisol 2 | 363.049 | 90.9 | 3.16 | 6 | 10 | 31 | 10 |
| Cortisol ^13^C_3_ 1 | 366.049 | 124.0 | 3.15 | 56 | 10 | 29 | 18 |
| Cortisol ^13^C_3_ 2 | 366.049 | 330.1 | 3.15 | 56 | 10 | 29 | 18 |
| Cortisone 1 | 361.030 | 163.0 | 2.81 | 145 | 10 | 46 | 11 |
| Cortisone 2 | 361.030 | 121.1 | 2.81 | 145 | 10 | 46 | 11 |
| Cortisone ^13^C_3_ 1 | 364.030 | 166.0 | 3.08 | 76 | 10 | 31 | 8 |
| Cortisone ^13^C_3_ 2 | 364.030 | 124.1 | 3.08 | 76 | 10 | 37 | 6 |
| Cortisone D_7_ | 368.030 | 169.0 | 2.75 | 145 | 10 | 46 | 11 |
| Progesterone 1 | 315.075 | 97.0 | 6.30 | 145 | 10 | 46 | 11 |
| Progesterone 2 | 315.075 | 108.9 | 6.30 | 145 | 10 | 46 | 11 |
| Progesterone ^13^C_3_ 1 | 318.952 | 100.0 | 6.30 | 26 | 10 | 29 | 16 |
| Progesterone ^13^C_3_ 2 | 318.952 | 112.1 | 6.30 | 26 | 10 | 31 | 12 |
| Progesterone D_9_ | 324.075 | 100.0 | 6.26 | 145 | 10 | 46 | 11 |
| Testosterone 1 | 288.874 | 97.0 | 5.15 | 145 | 10 | 46 | 11 |
| Testosterone 2 | 288.874 | 109.0 | 5.15 | 145 | 10 | 46 | 11 |
| Testosterone ^13^C_3_ 1 | 292.111 | 100.0 | 5.14 | 91 | 10 | 27 | 14 |
| Testosterone ^13^C_3_ 2 | 292.111 | 111.9 | 5.14 | 91 | 10 | 31 | 16 |

Table S4: The ratio of response of the surrogate and authentic analyte = RF.

| **Compound** | **RF** |
| --- | --- |
| D_5_-2-AG/ 2-AG | 1.91 |
| D_4_-AEA/ AEA | 1.13 |
| D_4_-OEA/ OEA | 5.83 |
| D_4_-PEA/ PEA | 2.24 |
| ^13^C_3_-cortisol/ Cortisol | 1.88 |
| ^13^C_3_-Cortisone/ Cortisone | 1.76 |
| ^13^C_3_-Androstenedione/ Androstenedione | 3.33 |
| ^13^C_3_-Progesterone/ Progesterone | 0.41 |
| ^13^C_3_-Testosterone/ Testosterone | 1.32 |

Table S5: Linearity and quantification limits for endocannabinoids and steroid hormones in nail. LOQ: Limit of Quantification, Linearity of the calibration was determined over the range pg/mg.

| **Compound** | **Calibration range**  **[pg/mg]** | | | **R^2^** |  | **LOQ**  **[pg/mg]** |
| --- | --- | --- | --- | --- | --- | --- |
| D_5_-2-AG | 5 | to | 200 | 0.99629 |  | 5 |
| D_4-_AEA | 0.1 | to | 10 | 0.99056 |  | 0.3 |
| D_4-_OEA | 20 | to | 10000 | 0.99740 |  | 5.0 |
| D_4-_PEA | 500 | to | 10000 | 0.99510 |  | 3.0 |
| ^13^C_3-_Cortisol | 0.3 | to | 50 | 0.99653 |  | 0.4 |
| ^13^C_3-_Cortisone | 1 | to | 500 | 0.99685 |  | 0.3 |
| ^13^C_3-_Androstenedione | 0.1 | to | 500 | 0.99944 |  | 0.3 |
| ^13^C_3-_Progesterone | 0.3 | to | 500 | 0.99819 |  | 0.5 |
| ^13^C_3-_Testosterone | 0.1 | to | 10 | 0.98973 |  | 0.3 |

Table S6: Validation parameters, accuracy (bias), precision, matrix effect and recovery for low, medium and high levels of endocannabinoids and steroid hormones. CV [%]:coefficient of variation percentage. Matrix effect of 100 % indicates no reduction or enhancement of signal. Over 100 % suggests enhancement of signal and below 100 % suggests suppression of signal.

| **Compound** | **Validation Conc.**  **[pg/mg]** | **Bias**  **[%]** | **Intra-day CV [%]** | **Inter-day CV [%]** | **Matrix Effect ± SD [%]** | **Recovery ± SD [%]** |
| --- | --- | --- | --- | --- | --- | --- |
| D_5_-2-AG | 10 | 5 | 7 | 7 | 167 ± 16 | 100 ± 24 |
|  | 50 | 5 | 4 | 11 |  |  |
|  | 150 | -5 | 4 | 6 | 165 ± 13 | 087 ± 70 |
| D_4_-AEA | 0.1 | -1 | 3 | 6 | 085 ± 12 | 095 ± 10 |
|  | 1 | 0 | 2 | 2 |  |  |
|  | 8 | 0 | 5 | 6 | 085 ± 70 | 085 ± 60 |
| D_4_-OEA | 600 | 19 | 2 | 17 | 122 ± 15 | 101 ± 12 |
|  | 2000 | 19 | 7 | 10 |  |  |
|  | 8000 | 10 | 3 | 3 | 104 ± 60 | 094 ± 60 |
| D_4_-PEA | 600 |  | 3 | 20 | 123 ± 17 | 088 ± 12 |
|  | 2000 | 12 | 5 | 14 |  |  |
|  | 8000 | 0 | 3 | 4 | 106 ± 16 | 086 ± 90 |
| ^13^C_3_-Cortisol | 0.6 | 7 | 3 | 3 | 076 ± 23 | 087 ± 15 |
|  | 5 | 7 | 3 | 4 |  |  |
|  | 40 | 6 | 3 | 4 | 80 ± 70 | 084 ± 20 |
| ^13^C_3_-Cortisone | 1.2 | 5 | 4 | 4 | 82 ± 50 | 081 ± 40 |
|  | 50 | 6 | 2 | 3 |  |  |
|  | 400 | 5 | 1 | 2 | 087 ± 80 | 084 ± 30 |
| ^13^C_3_-Androstenedione | 1.2 | 7 | 1 | 5 | 100 ± 50 | 087 ± 40 |
|  | 50 | 14 | 2 | 7 |  |  |
|  | 400 | 6 | 4 | 6 | 101 ± 50 | 092 ± 80 |
| ^13^C_3_-Progesterone | 1.2 | -6 | 5 | 7 | 087 ± 19 | 093 ± 11 |
|  | 50 | 10 | 3 | 2 |  |  |
|  | 400 | 11 | 1 | 2 | 078 ± 10 | 085 ± 50 |
| ^13^C_3_-Testosterone | 0.1 | 1 | 6 | 9 | 092 ± 10 | 083 ± 12 |
|  | 1 | 3 | 4 | 5 |  |  |
|  | 8 | 10 | 5 | 5 | 098 ± 50 | 088 ± 10 |

Tablele S7: Mean concentrations and relative standard deviation of six replicates of authentic nail pool

| **Compound** | **Mean [pg/mg]** | **RSD [%]** |
| --- | --- | --- |
| 2-AG | 64.6 | 12.1 |
| AEA | 1.9 | 2.4 |
| OEA | 3044.4 | 12.8 |
| PEA | 6218.2 | 20.2 |
| Cortisol | 210.4 | 4.4 |
| Cortisone | 5.2 | 2.4 |
| Androstenedione | 1.7 | 4.8 |
| Progesterone | 1.8 | 0.5 |
| Testosterone | 0.7 | 9.8 |

Table S8: Robustness Pool P17-602

| **N = 2** | **2-AG** | | **AEA** | | **OEA** | | **PEA** | | **Cortisol** | | **Cortisone** | | **Androstenedione** | | **Progesterone** | | | **Testosterone** | |
| --- | --- | --- | --- | --- | --- | --- | --- | --- | --- | --- | --- | --- | --- | --- | --- | --- | --- | --- | --- |
| **Weight [mg]** | **Mean [pg/mg]** |  | **Mean [pg/mg]** |  | **Mean [pg/mg]** |  | **Mean [pg/mg]** |  | **Mean [pg/mg]** |  | **Mean [pg/mg]** |  | **Mean [pg/mg]** |  | **Mean [pg/mg]** | |  | **Mean [pg/mg]** |  |
| 0.5 | 204.7 |  | 5.6 |  | 9778.8 |  | 12702.1 |  | 325.3 |  | 31.5 |  | - |  | 2.8 |  | | - |  |
| 1 | 118.9 |  | 3.0 |  | 5503.0 |  | 10764.7 |  | 256.5 |  | 19.2 |  | 10.5 |  | 2.8 |  | | 0.1 |  |
| 3 | 87.8 |  | 1.9 |  | 3209.9 |  | 10524.3 |  | 247.9 |  | 11.5 |  | 4.2 |  | 1.7 |  | | 0.5 |  |
| 5 | 76.8 |  | 1.7 |  | 3064.3 |  | 10490.0 |  | 238.8 |  | 11.0 |  | 2.8 |  | 1.6 |  | | 0.6 |  |
| 10 | 59.8 |  | 2.1 |  | 2656.3 |  | 8081.0 |  | 248.1 |  | 10.2 |  | 1.9 |  | 2.0 |  | | 0.7 |  |
| 20 | 56.0 |  | 1.5 |  | 2712.8 |  | 8942.5 |  | 187.2 |  | 9.0 |  | 2.2 |  | 2.2 |  | | 0.8 |  |
| **Mean** | 100.6 | | 2.6 | | 4487.5 | | 10250.8 | | 250.6 | | 15.4 | | 4.3 | | 2.2 | | | 0.5 | |
| **RSD [%]** | ± 53.12 | | ± 55.8 | | ± 59.6 | | ± 15.8 | | ± 18.2 | | ± 53.8 | | ± 78.0 | | ± 26.9 | | | ± 45.8 | |

Table S9: Robustness Pool P21-602

| **N = 2** | **2-AG** | | **AEA** | | **OEA** | | **PEA** | | **Cortisol** | | **Cortisone** | | **Androstenedione** | | **Progesterone** | | **Testosterone** | |
| --- | --- | --- | --- | --- | --- | --- | --- | --- | --- | --- | --- | --- | --- | --- | --- | --- | --- | --- |
| **Weight [mg]** | **Mean [pg/mg]** |  | **Mean [pg/mg]** |  | **Mean [pg/mg]** |  | **Mean [pg/mg]** |  | **Mean [pg/mg]** |  | **Mean [pg/mg]** |  | **Mean [pg/mg]** |  | **Mean [pg/mg]** |  | **Mean [pg/mg]** |  |
| 0.5 | 267.9 |  | 5.8 |  | 2387.9 |  | 3364.9 |  | 299.3 |  | 3.9 |  | - |  | 1.4 |  | - |  |
| 1 | 153.6 |  | 3.1 |  | 1767.6 |  | 5020.5 |  | 242.5 |  | 2.4 |  | 4.1 |  | 2.6 |  | - |  |
| 3 | 101.2 |  | 2.0 |  | 1791.7 |  | 6606.5 |  | 243.3 |  | 6.9 |  | 2.1 |  | 2.2 |  | 0.5 |  |
| 5 | 84.3 |  | 1.9 |  | 2072.2 |  | 6731.3 |  | 236.1 |  | 8.4 |  | 1.7 |  | 1.9 |  | 0.7 |  |
| 10 | 68.3 |  | 1.8 |  | 2014.5 |  | 5223.4 |  | 246.8 |  | 9.1 |  | 1.5 |  | 1.9 |  | 1.0 |  |
| 20 | 58.1 |  | 1.5 |  | 2217.8 |  | 5825.5 |  | 186.6 |  | 8.8 |  | 1.7 |  | 2.2 |  | 1.1 |  |
| **Mean** | 122.2 | | 2.7 | | 2042.0 | | 5462.0 | | 242.4 | | 6.6 | | 2.2 | | 2.0 | | 0.8 | |
| **RSD [%]** | ± 61.60 | | ± 57.72 | | ± 13.44 | | ± 23.67 | | ± 15.32 | | ± 42.84 | | ± 45.88 | | ± 20.11 | | ± 29.90 | |

Table S10: Robustness Pool P21-605

| **N = 2** | **2-AG** | | **AEA** | | **OEA** | | **PEA** | | **Cortisol** | | **Cortisone** | | **Androstenedione** | | **Progesterone** | | **Testosterone** | |
| --- | --- | --- | --- | --- | --- | --- | --- | --- | --- | --- | --- | --- | --- | --- | --- | --- | --- | --- |
| **Weight [mg]** | **Mean [pg/mg]** |  | **Mean [pg/mg]** |  | **Mean [pg/mg]** |  | **Mean [pg/mg]** |  | **Mean [pg/mg]** |  | **Mean [pg/mg]** |  | **Mean [pg/mg]** |  | **Mean [pg/mg]** |  | **Mean [pg/mg]** |  |
| 0.5 | 252.8 |  | 5.5 |  | 1626.6 |  | 822.1 |  | - |  | 3.0 |  | - |  | 0.6 |  | - |  |
| 1 | 146.5 |  | 3.5 |  | 1436.7 |  | 3103.5 |  | - |  | 8.6 |  | - |  | 0.9 |  | 0.6 |  |
| 3 | 68.9 |  | 6.72.3 |  | 1491.0 |  | 6431.7 |  | 0.4 |  | 11.8 |  | 1.6 |  | 0.8 |  | 2.4 |  |
| 5 | 57.8 |  | 2.5.67 |  | 1531.8 |  | 6967.2 |  | 0.6 |  | 12.0 |  | 1.2 |  | 0.7 |  | 2.2 |  |
| 10 | 83.0 |  | 2.66.1 |  | 2259.1 |  | 12055.8 |  | 1.8 |  | 12.7 |  | 1.4 |  | 0.8 |  | 2.7 |  |
| 20 | 116.0 |  | 2.610.3 |  | 3124.7 |  | 13148.6 |  | 2.9 |  | 12.3 |  | 1.2 |  | 0.8 |  | 3.1 |  |
| **Mean** | 120.8 | | 3.2 | | 1911.6 | | 7088.1 | | 1.4 | | 10.1 | | 1.4 | | 0.8 | | 2.2 | |
| **RSD [%]** | ± 57.40 | | ± 36.21 | | ± 33.54 | | ± 65.32 | | ± 76.96 | | ± 35.88 | | ± 12.78 | | ± 26.99 | | ± 41.99 | |

Table S11: Robustness Pool P21-607

| **N = 1** | **2-AG** | **AEA** | **OEA** | **PEA** | **Cortisol** | **Cortisone** | **Androstenedione** | **Progesterone** | **Testosterone** |
| --- | --- | --- | --- | --- | --- | --- | --- | --- | --- |
| **Weight**  **[mg]** | **[pg/mg]** | **[pg/mg]** | **[pg/mg]** | **[pg/mg]** | **[pg/mg]** | **[pg/mg]** | **[pg/mg]** | **[pg/mg]** | **[pg/mg]** |
| 3 | 60.2 | 3.6 | 604.9 | 1933.6 | - | 0.8 | - | 0.52 | - |
| 5 | 45.3 | 2.6 | 593.5 | 2145.4 | - | 1.7 | - | 0.41 | - |
| 10 | 43.3 | 3.0 | 597.0 | 2202.0 | - | 2.2 | 0.4 | 0.54 | - |
| 15 | 34.9 | 2.4 | 561.4 | 2223.2 | - | 2.1 | 0.3 | 0.53 | - |
| 20 | 35.4 | 2.5 | 611.1 | 2524.5 | 0.39 | 2.3 | 0.2 | 0.46 | - |
| 25 | 32.2 | 2.4 | 561.9 | 2273.7 | 0.37 | 2.6 | 0.3 | 0.51 | 0.03 |
| **Mean** | 41.9 | 2.8 | 588.3 | 2217.1 | 0.38 | 2.0 | 0.3 | 0.49 | - |
| **RSD [%]** | ± 24.66 | ± 16.23 | ± 3.66 | ± 8.64 | ± 3.66 | ± 22.27 | ± 25.33 | ± 10.15 | - |

Table S12: Robustness Pool P21-609

| **N = 1** | **2-AG** | **AEA** | **OEA** | **PEA** | **Cortisol** | **Cortisone** | **Androstenedione** | **Progesterone** | **Testosterone** |
| --- | --- | --- | --- | --- | --- | --- | --- | --- | --- |
| **Weight**  **[mg]** | **[pg/mg]** | **[pg/mg]** | **[pg/mg]** | **[pg/mg]** | **[pg/mg]** | **[pg/mg]** | **[pg/mg]** | **[pg/mg]** | **[pg/mg]** |
| 3 | 49.0 | 1.7 | 915.5 | 2867.1 | 0.1 | 16.8 | 1.3 | 1.0 | 0.3 |
| 5 | 36.5 | 1.1 | 859.7 | 2745.7 | 1.2 | 17.9 | 1.4 | 1.1 | 0.4 |
| 10 | 27.6 | 1.1 | 802.1 | 2729.6 | 2.2 | 16.7 | 1.1 | 1.0 | 0.4 |
| 15 | 26.4 | 0.9 | 847.1 | 3263.3 | 2.5 | 18.0 | 0.8 | 1.1 | 0.5 |
| 20 | 25.1 | 0.9 | 809.5 | 2468.2 | 2.5 | 17.3 | 0.7 | 1.1 | 0.5 |
| 25 | 23.6 | 0.8 | 828.7 | 3356.4 | 3.0 | 17.9 | 0.7 | 1.1 | 0.5 |
| **Mean** | 31.4 | 1.1 | 843.8 | 9071.7 | 1.9 | 17.4 | 1.0 | 1.1 | 0.4 |
| **RSD [%]** | ± 31.13 | ± 30.09 | ± 4.90 | ± 10.70 | ± 54.45 | ± 3.35 | ± 29.51 | ± 4.63 | ± 21.81 |

Table S13: Stability data: 6 aliquots of a QC low, medium and high, as well as an authentic pool were injected over the time of a batch series (24h). The variation of the measurements was calculated.

**Figures**


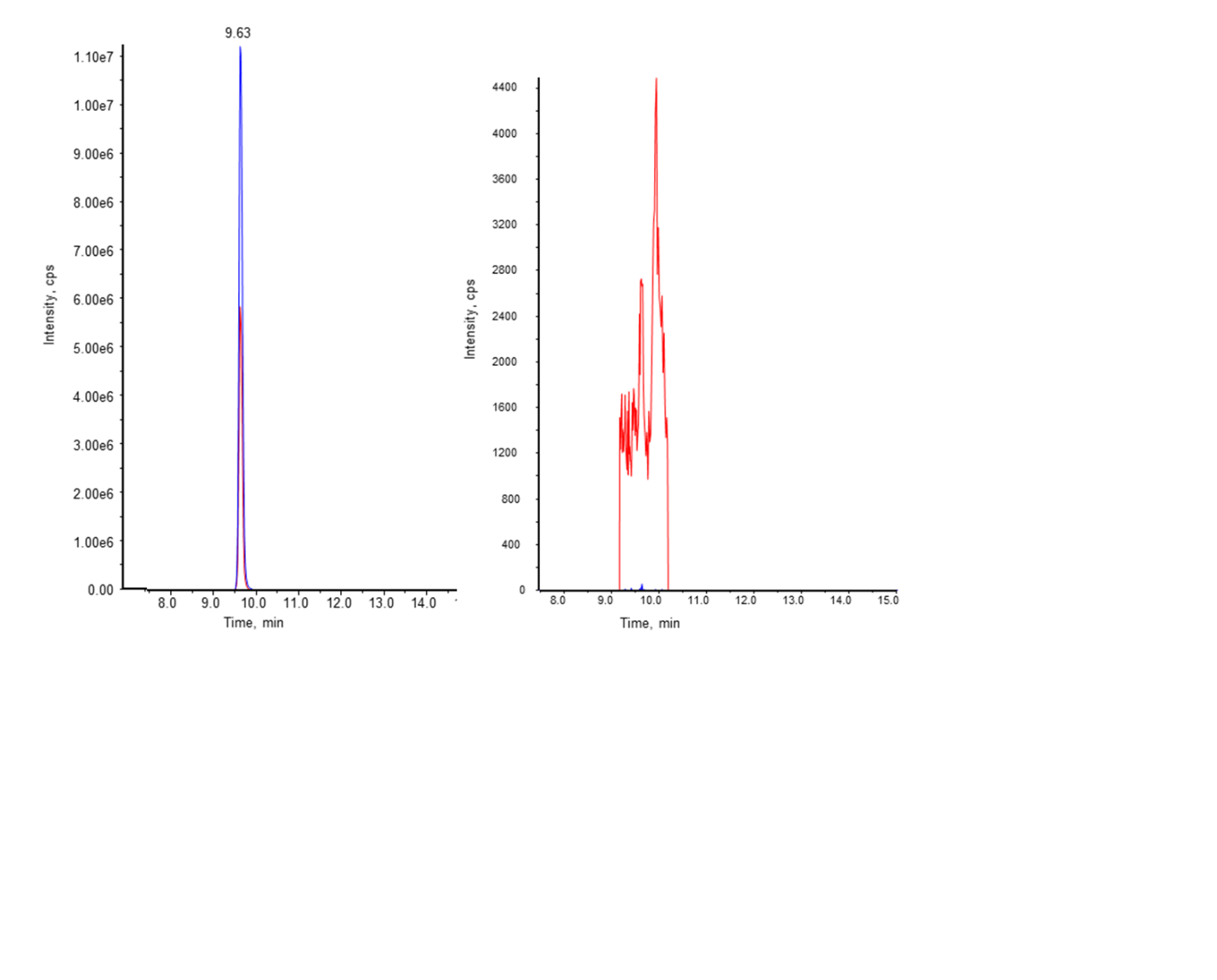


B

A

Figure S1: Graphic chromatogram as an example for the selectivity experiment (RT: 9.63 – transition 1 = blue and transition 2 = red) of AEA transitions. Peak A are transitions of AEA in a true positive sample of an AEA neat solution. Peak B are transitions of D4-AEA in a true positive sample of an AEA neat solution.

**
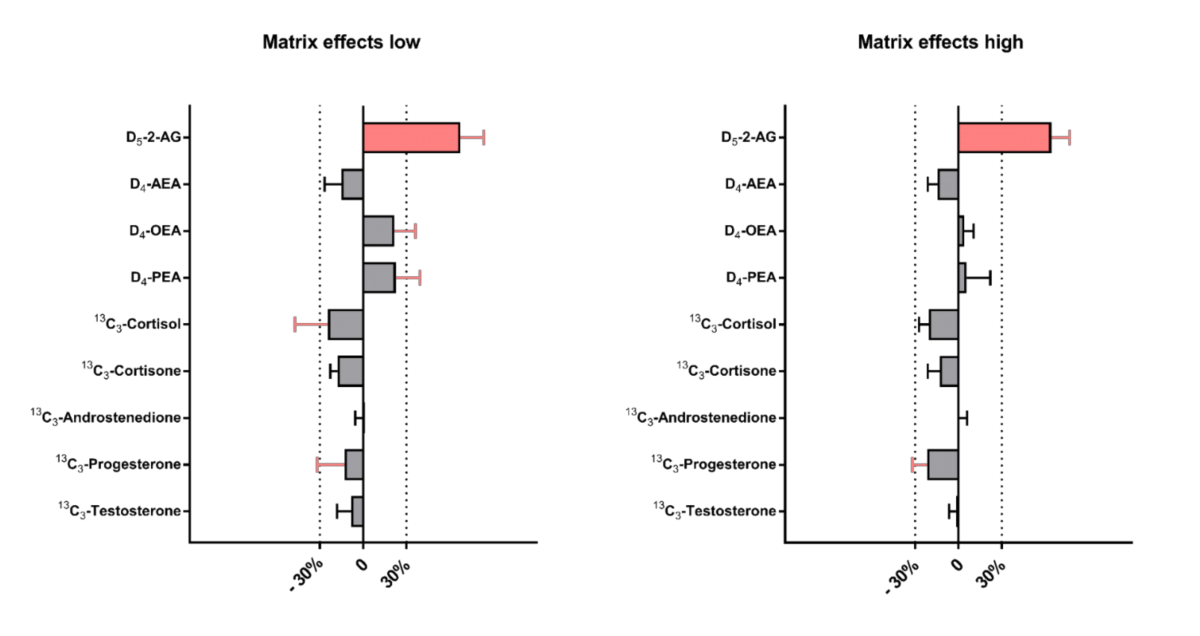
**

Figure S2: The graph illustrates the matrix effect of the endogenous analytes for QC low and QC high. The bars present the deviation of the matrix effects from 100 %. The dotted indicator lines on the x-axis at ± 30 % reflect the acceptance criteria for the matrix effects. The red marked bars (D_5_-2-AG) exceed the mentioned acceptance criteria. The error bars at the end of the bars represent the standard deviation of the evaluated matrix effects (n = 6).
